# ﻿Molecular phylogeny and morphology of the genus *Fuscoporia* (Hymenochaetales, Basidiomycota) and reveal three new species of the *F.ferrea* group

**DOI:** 10.3897/mycokeys.111.126446

**Published:** 2024-11-25

**Authors:** Qian Chen, Han Chen, Cheng-Hang Luo, Xiao-Hong Lai

**Affiliations:** 1 College of Architecture and Urban Planning, Chongqing Jiaotong University, Chongqing 400074, China; 2 College of Architecture and Urban Planning, Tongji University, Shanghai 200092, China; 3 Key Laboratory of Ecology and Energy-saving Study of Dense Habitat (Tongji University), Ministry of Education, Tongji University, Shanghai 200092, China

**Keywords:** Hymenochaetaceae, phylogeny, polypore, taxonomy

## Abstract

*Fuscoporia* is a polypore genus of Hymenochaetaceae that causes wood decay, although some species in the genus have medicinal values. Phylogenetic analyses of concatenated ITS1-5.8S-ITS2-nLSU sequence data and morphological features identified three new species, *F.eucalypticola*, *F.resupinata* and *F.subtropica* from Australia, China and Malaysia, and these new species derived from the *Fuscoporiaferrea* group. These three species are illustrated and described. A key to resupinate species of *Fuscoporia* without mycelial setae in the world is provided.

## ﻿Introduction

The genus *Fuscoporia* Murrill (Hymenochaetales, Basidiomycota) with *F.ferruginosa* (Schrad.) Murrill as the type species was first described in 1907 (Murrill 1907). For a long time, it has been considered as a synonym of *Phellinus* Quél. *sensu lato* (Gilbertson 1979; Larsen and Cobb-Poulle 1990; Ryvarden and Gilbertson 1994; Ryvarden 2004). Phylogenetic studies confirmed that the currently recognized genus of *Fuscoporia* is monophyletic (Wagner and Fischer 2001, 2002; Wu et al. 2022a). *Fuscoporia* is characterized by an almost light to dark brown, resupinate, effused-reflexed to pileate basidiomata, dimitic hyphal system with generative hyphae bearing crystals, presence of hymenial setae in most species, and hyaline, thin-walled, smooth basidiospores (Fiasson and Niemelä 1984; Chen et al. 2020; Wu et al. 2022a). The species is very rich and 104 *Fuscoporia* species have been recognized (Wu et al. 2022a, b; Chen et al. 2023a, b; https://www.mycobank.org; accessed on 08-9-2024). Among them, 62 species were described during the last five years (Chen and Dai 2019; Chen et al. 2019, 2020, 2022, 2023a, b; Du et al. 2020; Tchoumi et al. 2020; Vlasák et al. 2020; Yuan et al. 2020; Raymundo 2021; Hussain et al. 2022; Wu et al. 2022a; Cho et al. 2023; Olou et al. 2023; Bittencourt et al. 2024).

Some species of *Fuscoporia* are difficult to identify because most morphological features of these species overlap. The recent studies revealed some traditional species of *Fuscoporia* are actually the species complex, such as, *Fuscoporiacontigua* (Pers.) G. Cunn. (Cunningham 1948) was considered as a single species with variable basidiospores (oblong-ellipsoid or cylindric; Niemelä 2005; Dai 2010; Ryvarden and Melo 2017), but two Asian species were derived from *F.contigua* (Chen et al. 2019). A more comprehensive study (Chen et al. 2020, 2022; Wu et al. 2022a) of the genus revealed *Fuscoporiacontigua* is actually the most complex speciesin that its members do not share a common geographic distribution and host preference. So far, fourteen species have been published in *F.contigua* group. The taxonomic status of some species in the genus *Fuscoporia* is in need of re-evaluation.

*Fuscoporiaferrea* (Pers.) G. Cunn. (1948) was characterized by resupinate, annual to perennial basidiomata, cylindric spores and distribution in the Northern Hemisphere (Cunningham 1948; Ryvarden and Gilbertson 1994; Lowe 1966). Based on time divergence, the early divergence of the *Fuscoporia* species was inferred to occur in subtropics of southern Asia with a resupinate fruiting body, and *Fuscoporiaferrea* group is the oldest lineage in the genus with stem age estimated around 49.52 Myr (Hussain et al. 2022). Some Asian specimens previously identified as *Fuscoporiaferrea* were confirmed as different species based on morphological examinations and phylogenetic analyses, and described as *F.ramulicola* Y.C. Dai & Q. Chen, *F.subferrea* Q. Chen & Y. Yuan and *F.yunnanensis* Y.C. Dai (Dai 2010; Chen and Yuan 2017; Chen and Dai 2019). In addition, *F.punctatiformis* (Murrill) Zmitr., Malysheva & Spirin was combined (Spirin et al. 2006). So five species comprise the *F.ferrea* complex and are characterized by resupinate basidiomata, absence of mycelial setae, presence of hymenial setae and cystidioles, and cylindric basidiospores (Chen et al. 2020).

In the process of exploring of wood-decaying fungi, brown and resupinate specimens were collected from southern Asia and Australia, and their morphological characteristics, taxonomic relationships and phylogenetic affinities were analyzed. Three new taxa were confirmed within *Fuscoporiaferrea* group, and they are described and illustrated. A key to resupinate and mycelial setaeless species of *Fuscoporia* in the world is provided.

## ﻿Materials and methods

### ﻿Morphological studies

The research specimens are conserved in the herbarium of Microbiology, Beijing Forestry University (BJFC). The macroscopic color codes follow Petersen (1996) and the microscopic analyses follow Wang et al. (2023, 2024) and Zhao et al. (2023, 2024) using a Nikon Eclipse 80i microscope with phase contrast illumination. Samples for microscopic examination and drawings were prepared from slides stained with Cotton Blue follow Zhang et al. (2023). The following abbreviations are used: **CB–** = acyanophilous, **IKI–** = neither amyloid nor dextrinoid, **L** = mean length of all spore, **W** = mean width of all spore, **Q** = L/W ratios, **n (a/b)** = number of measured spores(a) form number of specimens (b).

### ﻿Molecular methods

According to the manufacturer’s instructions, a CTAB rapid plant genome extraction kit (Aidlab Biotechnologies Co., Ltd., Beijing, China) was used to obtain PCR products from dried samples. To generate PCR amplicons, the following primer pairs were used: ITS4 and ITS5 for the ITS1-5.8S-ITS2 region (White et al. 1990), LR0R and LR7 for the nLSU region (Vilgalys and Hester 1990). The PCR procedure was as follows: for the ITS1-5.8S-ITS2 region initial denaturation at 95 °C for 3 min, followed by 35 cycles at 94 °C for 40 s, 54 °C for 45 s and 72 °C for 1 min, and a final extension of 72 °C for 10 min; for nLSU, initial denaturation at 94 °C for 1 min, followed by 35 cycles at 94 °C for 1 min, 50 °C for 1 min and 72 °C for 1.5 min, and a final extension of 72 °C for 10 min. The PCR products were purified and sequenced by the Beijing Genomics Institute with the same primers. All newly generated sequences were deposited at GenBank and listed in Table 1 (http://www.ncbi.nlm.nih.gov/genbank). Besides the newly generated sequences for this study, other related sequences downloaded from GenBank based on Chen et al. (2023b) and Wu et al. (2022a, b) to explore the phylogenetic position of the newly sequenced specimens in *Fuscoporia*.

**Table 1.** Species name, specimens, origin and GenBank accession number of sequences used in this study.

Species name	Specimens	Origin	GenBank accession no.	
			ITS	nLSU
*Fuscoporiaacutimarginata*	Dai 15137	China	MH050751	MH050765
*F.acutimarginata*	Dai 16892	China	MH050752	MH050766
*F.ambigua*	Cui 9244	China	MN816706	MN809995
*F.ambigua*	JV 0509/151	USA	MN816707	MN809996
*F.americana*	JV 1209/3-J	USA	–	MG008466
*F.americana*	JV 1209/100	USA	KJ940022	MG008467
*F.atlantica*	SP 445618	Brazil	KP058515	KP058517
*F.atlantica*	SP 465829	Brazil	KP058514	KP058516
*F.australasica*	Dai 15625	China	MN816726	MN810018
*F.australasica*	Dai 15636	China	MG008397	MG008450
*F.australiana*	Dai 18672	Australia	MN816703	MN810014
*F.australiana*	Dai 18879	Australia	MN816705	MN810015
*F.bambusae*	Dai 16599	Thailand	MN816711	MN809999
*F.bambusae*	Dai 16615	Thailand	MN816715	MN810001
*F.caymanensis*	JV 1908/74	French Guiana	MT676832	MT676833
*F.caymanensis*	JV 1408/5	Costa Rica	MW009110	MW009109
*F.callimorpha*	Dai 17388	Brazil	MN121765	MN121824
*F.callimorpha*	Doll 868	Unknown	MN816701	MN809992
*F.chinensis*	Dai 15713	China	MN816721	MN810008
*F.chinensis*	Cui 11209	China	MN121767	MN121826
*F.chrysea*	JV 1607/106	Costa Rica	MN816736	MN810027
*F.centroamericana*	JV 1607/93	Costa Rica	MG008444	MG008460
*F.centroamericana*	O 908267	Costa Rica	MG008443	–
*F.contigua*	Dai 16025	USA	MG008401	MG008454
*F.contigua*	Dai 13567A	Romania	MG008402	MG008455
*F.costaricana*	JV 1407/92	Costa Rica	MG008446	MG008461
*F.costaricana*	JV 1504/85	Costa Rica	MG008413	MG478454
*F.dhofarensis*	ATN-007	Oman	OP593104	OP593105
*F.dolichoseta*	SFC20191015-23	Korea	ON427765	ON427795
*F.dolichoseta*	SFC20161006-16	Korea	ON427789	ON427817
*F.eucalypti*	Dai 18783	Australia	MN816730	MN810021
*F.eucalypti*	Dai 18792	Australia	MN816731	MN810022
***F.eucalypticola***	**Dai 18592A**	**Australia**	**PP732562**	**PP732631**
***F.eucalypticola***	**Dai 18683**	**Australia**	**PP732563**	**PP732632**
*F.ferrea*	MUCL 45984	France	KX961112	KY189112
*F.ferrea*	Cui 11801	China	KX961101	KY189101
*F.ferruginosa*	JV 0408/28	Czech Republic	KX961103	KY189103
*F.ferruginosa*	Dai 13200	France	MN816702	MN809993
*F.gilva*	JV 0709/75	USA	MN816720	MN810007
*F.gilva*	JV 1209/65	USA	MN816719	MN810006
*gilva*	URM 83957	Brazil	MH392545	MH407344
*gilvoides*	SFC2018042‐12	Korea	ON427763	ON427793
*gilvoides*	MUGBt	Pakistan	ON427781	ON427810
*F.hainanensis*	Dai 16105	China	–	ON520809
*F.hainanensis*	Dai 16110	China	–	ON520810
*F.hawaiana*	JV 2208/H-22-J	USA	OQ817709	OQ817855
*F.hawaiana*	JV 2208/H-30-J	USA	OQ817710	OQ817856
*F.insolita*	Spirin 5251	Russia	KJ677113	–
*F.insolita*	Spirin 5208	Russia	MN816724	MN810016
*F.karsteniana*	Dai 16552	China	MN816716	MN810002
*F.karsteniana*	Dai 11403	China	MN816717	MN810003
*F.kenyana*	Dai 19205	Kenya	OP580527	OP580521
*F.kenyana*	Dai 19202	Kenya	OP580526	OP580520
*F.koreana*	SFC20150625-05	Korea	ON427776	ON427805
*F.koreana*	SFC20160726-93	Korea	ON427762	ON427792
*F.latispora*	JV 1109/48	USA	MG008439	MG008468
*F.latispora*	JV 0610/VII-Kout	Mexico	MG008436	MG008469
*F.licnoides*	URM 84107	Brazil	MH392556	MH407355
*F.licnoides*	URM 83001	Brazil	MH392561	MH407357
*F.marquesiana*	URM 83094	Brazil	MH392544	MH407343
*F.minutissima*	JV 2208/H12-J	USA	OQ817711	OQ817857
*F.minutissima*	JV 2208/H16-J	USA	OQ817712	OQ817858
*F.monticola*	Dai 10909	China	MG008410	–
*F.monticola*	Dai 11860	China	MG008406	MG008457
*F.palomari*	JV 1004/5-J	USA	MN816737	–
*F.palomari*	JV 1305/3-J	USA	MN816738	MN810028
*F.plumeriae*	Dai 17814	Singapore	MN816714	MN810011
*F.plumeriae*	Dai 18858	Australia	MN816712	MN810010
*F.punctatiformis*	Dai 17443	Brazil	MH050755	MH050764
*F.punctatiformis*	Doll#872a	Brazil	MH050753	–
*F.pulviniformis*	CMW 48060	South Africa	MH599101	MH599125
*F.pulviniformis*	CMW 48600	South Africa	MH599102	MH599127
*F.ramulicola*	Dai 15723	China	MH050749	MH050762
*F.ramulicola*	Dai 16155	China	MH050750	MH050763
*F.reticulata*	SFC20121010-19	Korea	ON427766	–
*F.reticulata*	SFC20160115-16	Korea	ON427761	ON427791
*F.rhabarbarina*	Dai 16550	China	MN816744	MN810036
*F.rhabarbarina*	Dai 16226	China	MN816743	MN810035
***F.resupinata***	**Dai 20455**	**China**	**PP732567**	**PP732636**
***F.resupinata***	**Dai 20422**	**China**	**PP732568**	**PP732637**
***F.resupinata***	**Dai 21201**	**Malaysia**	**PP732569**	**PP732638**
*F.roseocinerea*	JV 1407/84	Costa Rica	MN816740	MN810030
*F.roseocinerea*	JV 1109/78-J	USA	MN816742	MN810032
*F.rufitincta*	JV 1008/25	USA	KJ940029	KX058575
*F.rufitincta*	JV 0904/142	USA	KJ940030	KX058574
*F.sarcites*	JV 0402/20K	Venezuela	MZ264225	MZ264218
*F.scruposa*	CMW 48145	South Africa	MH599105	MH599130
*F.scruposa*	CMW 47749	South Africa	MH599106	MH599129
*F.semiarida*	URM 83800	Brazil	MH392562	MH407361
*F.semiarida*	URM 82510	Brazil	MH392563	MH407362
*F.semicephala*	SFC20170524-08	Korea	ON427764	ON427794
*F.semicephala*	SFC20170712-20	Korea	ON427787	ON427815
*F.senex*	MEL 2382630	Australia	KP012992	–
*F.senex*	KAUNP MK41	Sri Lanka	KP794600	–
*F.septoseta*	Dai 12820	USA	MG008405	MN810033
*F.septoseta*	JV 0509/78	USA	MG008404	–
*F.setifera*	Dai 15710	China	MH050758	MH050767
*F.setifera*	Dai 15706	China	MH050759	MH050769
*F.shoreae*	Dai 17806	Singapore	MN816734	MN810025
*F.shoreae*	Dai 17818	Singapore	MN816735	MN810026
*F.sinica*	Dai 15468	China	MG008412	MG008459
*F.sinica*	Dai 15489	China	MG008407	MG008458
*F.sinuosa*	Dai 20498	China	MZ264226	MZ264219
*F.sinuosa*	Dai 20499	China	MZ264227	MZ264220
*F.subchrysea*	Dai 16201	China	MN816708	MN809997
*F.subchrysea*	Dai 17656	China	MN816709	MN809998
*F.subferrea*	Dai 16326	China	KX961097	KY053472
*F.subferrea*	Dai 16327	China	KX961098	KY053473
*F.submurina*	Dai 19501	Sri Lanka	MZ264229	MZ264222
*F.submurina*	Dai 19655	Sri Lanka	MZ264228	MZ264221
***F.subtropica***	**Dai 20476**	**China**	**PP732564**	**PP732633**
***F.subtropica***	**Dai 19957**	**China**	**PP732565**	**PP732634**
***F.subtropica***	**Dai 22604**	**China**	**PP732566**	**PP732635**
*F.torulosa*	JV 1405/2	Czech Republic	KX961106	KY189106
*F.torulosa*	Dai 15518	China	MN816732	MN810023
*F.viticola*	JV 0911/6	Czech Republic	KX961110	–
*F.viticola*	He 2123	USA	MN816725	MN810017
*F.wahlbergii*	JV 1312/20-Kout	Spain	MN816727	MG008462
*F.wahlbergii*	JV 0709/169-J	USA	MN816728	–
*F.yunnanensis*	Cui 8182	China	MH050756	MN810029
*F.yunnanensis*	Dai 15637	China	MH050757	MH050768
**Outgroups**				
*Coniferiporiaweirii*	CFS 504	Canada	AY829341	AY829345
*Phellinidiumfragrans*	CBS 202.90	USA	AY558619	AY05027

### ﻿Phylogenetic analysis

The following software was used for data processing and phylogenetic analysis: BioEdit (Hall 1999), ClustalX (Thompson et al. 1997) and MAFFT (http://mafft.cbrc.jp/alignment/server/, Katoh et al. 2017) for sequences and manually adjusted, PhyloSuite v.1.2.2 (Zhang et al. 2020) for concatenated the separate alignments, PAUP* 4.0b10 (Swofford 2002) for maximum parsimony (MP) analysis, raxmlGUI 1.2 (Silvestro and Michalak 2012) for maximum likelihood (ML) analysis and MrBayes 3.2.6 (Ronquist and Huelsenbeck 2003) for Besian Inference (BI), TreeView 1.5.0 and FigTree version 1.4.4 (Rambaut 2018) to show the phylogenetic tree. The best topologies from ML analyses are shown in this study and the final alignments and the retrieved topologies has been deposited at TreeBASE (http://treebase.org/treebase-web/home.html), study ID: 31700.

In Maximum likelihood (ML) methods, statistical support values were obtained by using nonparametric bootstrapping with 1000 replicates, with default settings for all parameters. For BI analysis, the best-fit partitioning scheme and substitution model were determined by using ModelFinder (Kalyaanamoorthy et al. 2017). Tree was sampled every 1000 generations, starting from random trees with four chains for 2.5 million generations. In maximum parsimony (MP) analysis, tree was inferred using the heuristic search option with tree bisection reconnection (TBR) branch swapping and 1000 random sequence additions. The maxtrees parameter was set to 5000, branches of zero length were collapsed, and all parsimonious trees were saved. Clade robustness was assessed by a bootstrap analysis with 1000 replicates (Felsenstein 1985). Descriptive tree statistics such as tree length (TL), consistency index (CI), retention index (RI), rescaled consistency index (RC), and homoplasy index (HI), were calculated. The three phylogenetic methods produced a similar topology for each dataset, so, only the topology of the ML tree is presented along. Branches that received bootstrap support for ML and MP not less than 75% and BPP not less than 0.95 were considered as significantly supported.

## ﻿Results

### ﻿Molecular phylogeny

**Figure 1.**
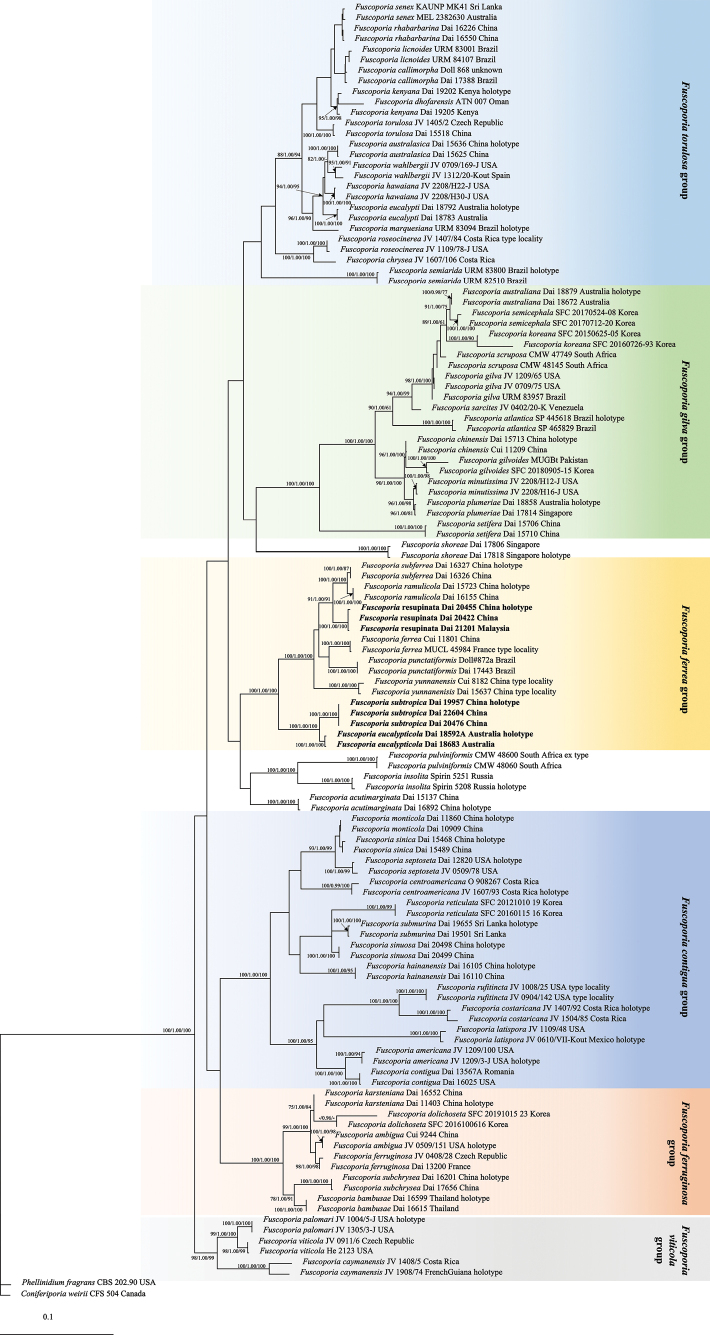
Maximum Likelihood (ML) tree illustrating the phylogeny of *Fuscoporia* and related species generated inferred from a combined ITS1-5.8S-ITS2-nLSU dataset. Statistical values (ML//BI/MP) are indicated for each node that received bootstrap support from ML and MP ≥ 75% and BPP ≥ 0.90. Names of new species are in bolds.

In this study, the data set of ITS and nLSU region included 118 ITS and 110 nLSU sequences from 121 samples, representing 61 species of *Fuscoporia* and *Coniferiporiaweirii* (Murrill) L.W. Zhou & Y.C. Dai and *Phellinidiumfragrans* (M.J. Larsen & Lombard) Nuss as the outgroups (Table 1, Fig. 1) based on previous studies (Chen and Yuan 2017). The dataset had an aligned length of 2224 characters, of which 1392 were constant, 120 variable but parsimony-uninformative, and 712 parsimony-informative. MP analysis yielded four similar topologies (TL = 3361, CI = 0.406, RI = 0.840, RC = 0.341, HI = 0.594). The BI analysis resulted in a concordant topology with an average standard deviation of split frequencies of 0.002648. The best model suggested by MrModeltest and applied in Bayesian analysis was GTR+F+I+G4 for ITS1+ITS2, K2P for 5.8s and K2P+I+G4 for nLSU. MP and BI analysis also resulted in a topology similar to that of the ML analysis. The seven specimens formed three lineages, named as *Fuscoporiaeucalypticola*, *F.resupinata* and *F.subtropica*, with high support (100 in ML/1.00 in BI/100 in MP, respectively), which clustered together with *F.ferrea*, *F.punctatiformis*, *F.ramulicola*, *F.subferrea* and *F.yunnanensis*, in the *F.ferrea* clade with strongly support (100 in ML/1.00 in BI/100 in MP).

### ﻿Taxonomy

#### ﻿ Fuscoporia eucalypticola

Taxon classificationFungiHymenochaetalesHymenochaetaceae

Q. Chen sp. nov.

71E27897-E5B5-5866-AFC7-653C97D5BD4E

853957

Figs 2 , 3

##### Holotype.

Australia • Victoria, Yarra Ranges National Park, on fallen branch of *Eucalyptus*, 9 May 2018, Dai 18592A (BJFC 027061).

##### Etymology.

*Eucalypticola* (Lat.): refere to the species growing on *Eucalyptus*.

##### Description.

***Basidiomata*.** Annual, resupinate, inseparable from the substrate, without odor or taste and corky when fresh, rigid when dry, up to 20 cm long, 3 cm wide and 1.5 mm thick at center. Pore olivaceous buff to greyish brown; sterile margin narrow or almost lacking, buff, up to 1 mm wide; pores irregular or sinuous, 3–5 per mm; dissepiments thin, entire, abundant setae seen in tube cavities (under lens). Subiculum clay-buff, corky, about 0.1 mm thick. Tubes olivaceous buff, up to 1 mm long.

**Figure 2.**
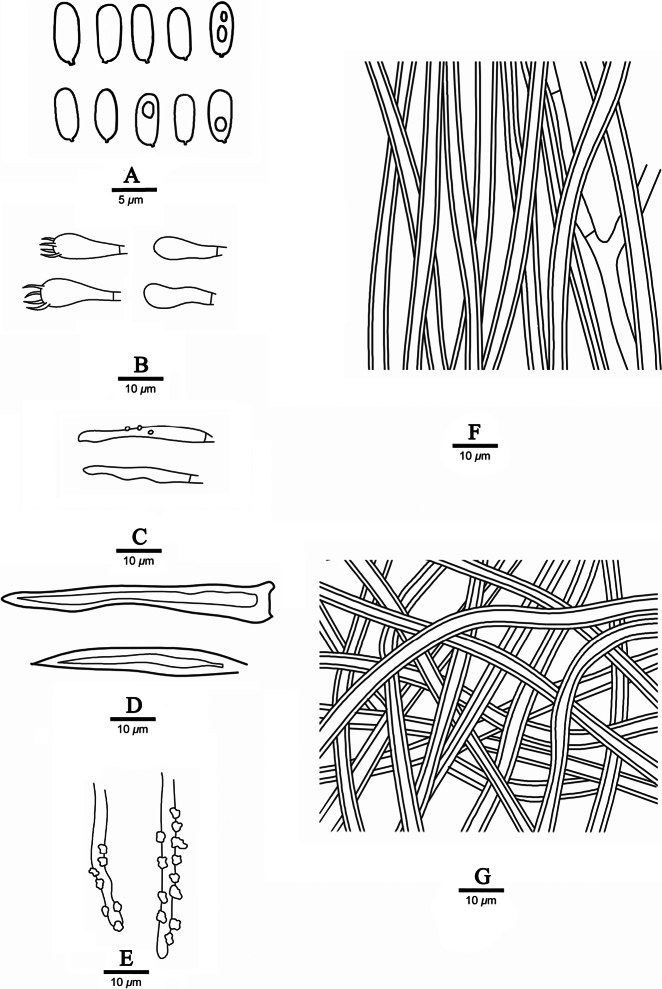
Microscopic structures of *Fuscoporiaeucalypticola* (Dai 18592A, holotype) **A** basidiospores **B** basidia and basidioles **C** cystidioles **D** hymenial setae **E** generative hyphae at dissepiment edge **F** hyphae from tube trama **G** hyphae from subiculum.

***Hyphal structure*.** Hyphal system dimitic; generative hyphae simple septate; tissue becoming black in KOH.

***Subiculum*.** Generative hyphae infrequently, thin-walled, frequently branched, simple septate, 1.5–2.5 μm in diam; skeletal hyphae dominant, yellowish brown, thick-walled with a medium lumen, unbranched, aseptate, flexuous, strongly interwoven, 2–3 μm in diam.

**Figure 3.**
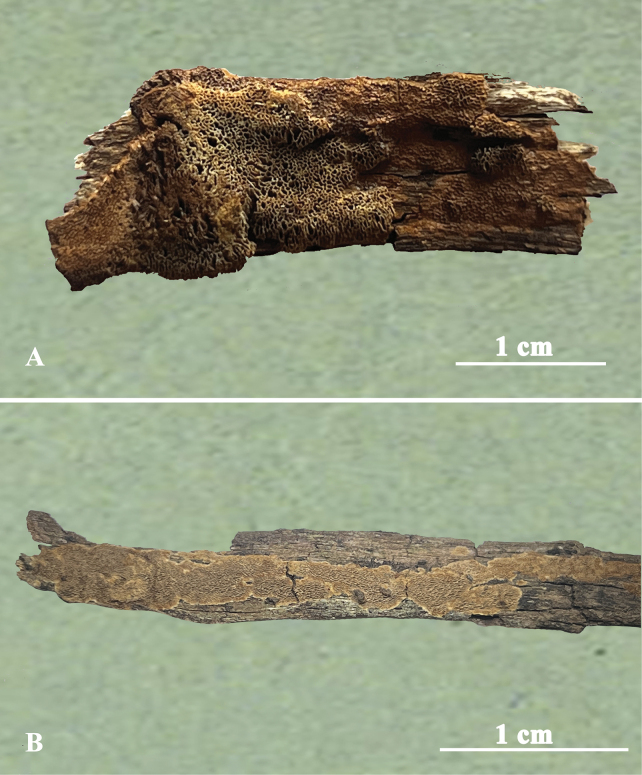
Basidiomata of *Fuscoporiaeucalypticola***A** Dai 18592A (holotype) **B** Dai 18683.

***Tubes*.** Generative hyphae infrequent, mostly present at subhymenium, hyaline, thin-walled, frequently branched, simple septate, 1.5–2.5 μm in diam; skeletal hyphae dominant, yellowish brown, thick-walled with a wide lumen, unbranched, aseptate, loosely interwoven to subparallel along the tubes, 2–3 μm in diam. Setae frequent, mostly originating from hymenium, subulate, dark brown, thick-walled, 30–60 × 4–6 μm; fusoid cystidioles frequent, hyaline and thin-walled, 25–32 × 2–4 μm; basidia barrel-shaped, with four sterigmata and a simple septum at the base, 20–25 × 4–7 μm; basidioles in shape similar to basidia, but slightly smaller.

***Basidiospores*.** Basidiospores cylindric, hyaline, thin-walled, smooth, IKI–, CB–, sometimes with a small guttule, 6.2–8 × (2–)2.1–3 μm, L = 7.03 μm, W = 2.37 μm, Q = 2.86–3.05 (n = 60/2).

##### Additional specimen examined.

Australia • Melbourne, Dandenong Ranges Botanical Garden, on fallen branch of *Eucalyptus*, 12 May 2018, Dai 18683 (BJFC 027152).

#### ﻿ Fuscoporia resupinata

Taxon classificationFungiHymenochaetalesHymenochaetaceae

Q. Chen sp. nov.

CB2B7701-BA9D-52D0-964E-CFA1E7D0C088

853956

Figs 4 , 5

##### Holotype.

China • Yunnan Province, Pu’er, Taiyanghe National Forest Park, on dead angiosperm tree, 17 August 2019, Dai 20455 (BJFC032123).

##### Etymology.

Resupinata (Lat.): refers to the species having resupinate basidiomata.

##### Description.

***Basidiomata*.** Annual, resupinate, inseparable from the substrate, without odor or taste and corky when fresh, rigid when dry, up to 10.6 cm long, 4 cm wide and 1.2 mm thick at center. Pore surface fawn when fresh, snuff brown when dry; sterile margin indistinct, honey-yellow when dry, up to 1 mm wide, paler than color than the pore surface; pores circular to angular, 5–7 per mm; dissepiments thin, entire, abundant setae seen in tube cavities (under lens). Subiculum honey yellow, corky, about 0.2 mm thick. Tubes grayish brown, paler contrasting with pores, rigid, up to 1 mm long.

**Figure 4.**
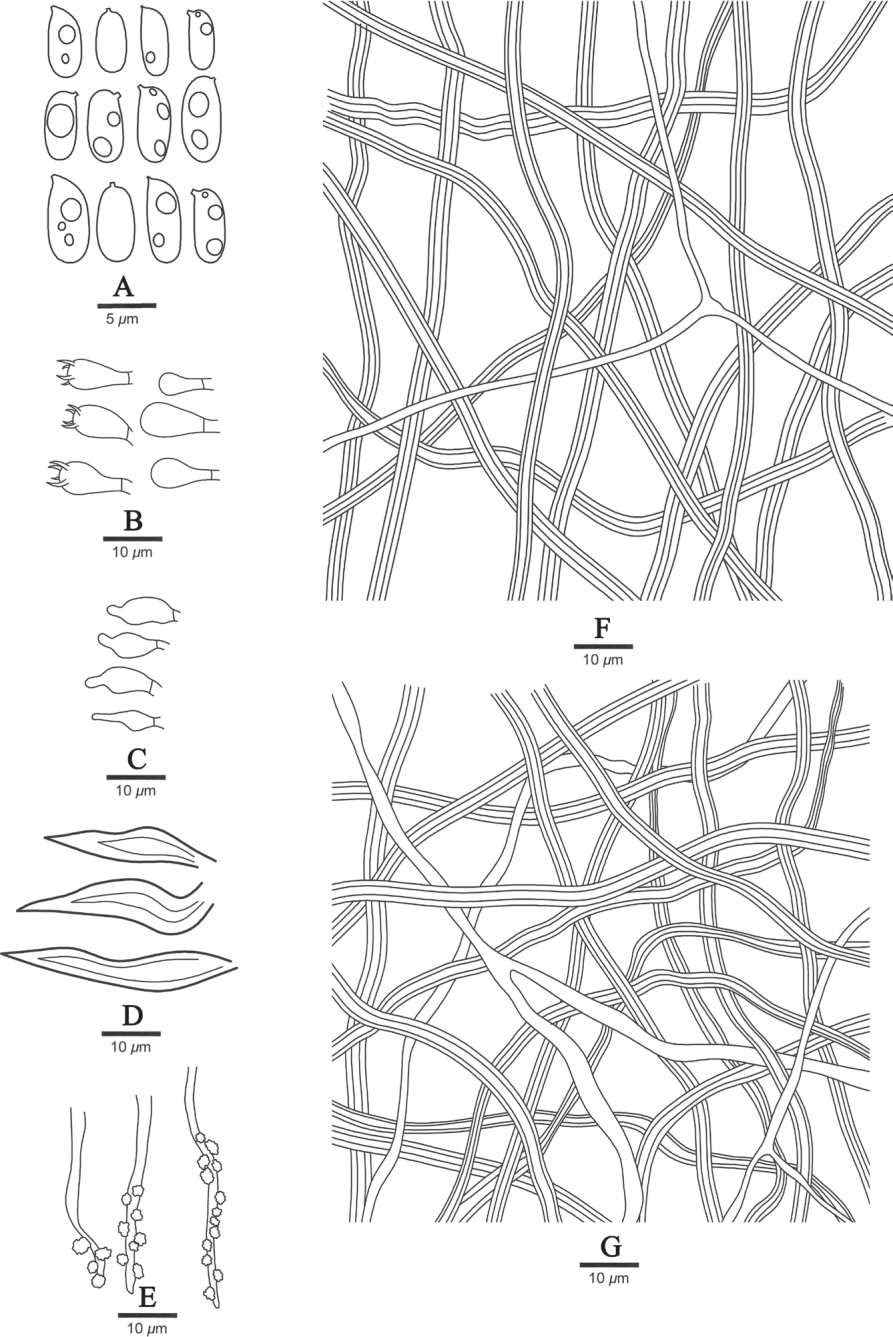
Microscopic structures of *Fuscoporiaresupinata* (holotype, Dai 20455) **A** basidiospores **B** basidia and basidioles **C** cystidioles **D** hymenial setae **E** generative hyphae at dissepiment edge **F** hyphae from tube trama **G** hyphae from subiculum.

***Hyphal structure*.** Hyphal system dimitic; generative hyphae simple septate; tissue becoming black in KOH.

***Subiculum*.** Generative hyphae infrequently, thin-walled, frequently branched, simple septate, 1–1.5 μm in diam; skeletal hyphae dominant, yellowish brown, thick-walled with a narrow to medium lumen, unbranched, aseptate, flexuous, strongly interwoven, 3–4 μm in diam.

**Figure 5.**
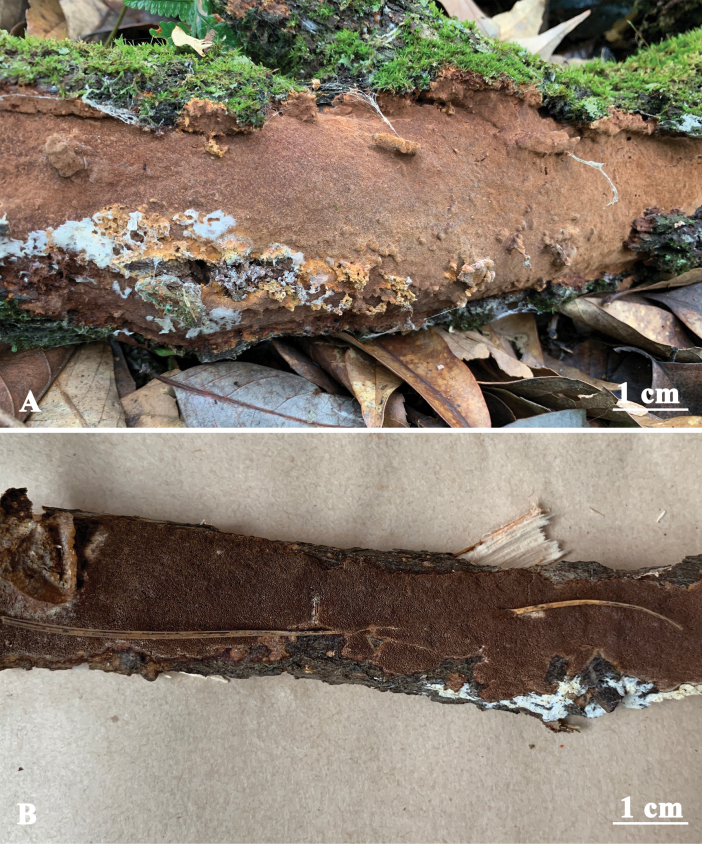
Basidiomata of *Fuscoporiaresupinata***A** Dai 20455 (holotype) **B** Dai 20422.

***Tubes*.** Generative hyphae infrequent, mostly present at subhymenium, hyaline, thin-walled, frequently branched, simple septate, 1–2 μm in diam; skeletal hyphae dominant, yellowish brown, thick-walled with a narrow to medium lumen, unbranched, aseptate, loosely interwoven, 2–5 μm in diam. Setae frequent, mostly originating from hymenium, subulate, dark brown, thick-walled, 20–30 × 5–7 μm; fusoid cystidioles frequent, hyaline and thin-walled, sometimes covered with crystals, 8–12 × 3.5–5 μm; basidia barrel-shaped, with four sterigmata and a simple septum at the base, 16–20 × 6–8 μm; basidioles in shape similar to basidia, but slightly smaller.

***Basidiospores*.** Basidiospores cylindric, hyaline, thin-walled, smooth, usually glued in tetrads, IKI–, CB–, sometimes with guttules, (5.4–)5.5–7(–7.2) × (2.4–)2.5–3 μm, L = 6.38 μm, W = 2.71 μm, Q = 2.29–2.44 (n = 60/2).

##### Additional specimens examined.

China • Yunnan Province, Xinping County, Longquan Park, on fallen angiosperm branch, 16 August 2019, Dai 20422 (BJFC 032090). Malaysia • Selangor, Kota Damansara, Community Forest Reserve, on dead angiosperm tree, 7 December 2019, Dai 21201 (BJFC 032855).

#### ﻿ Fuscoporia subtropica

Taxon classificationFungiHymenochaetalesHymenochaetaceae

Q. Chen sp. nov.

9B833DED-7327-57F6-9D9E-A1CAC10A77DC

853958

Figs 6 , 7

##### Holotype.

China • Yunnan Province, Wenshan Zhuang and Miao Autonomous Region, Xichou County, Xiaoqiaogou Forest Farm, on fallen angiosperm trunk, 29 June 2019, Dai 19957 (BJFC 031631).

##### Etymology.

*Subtropica* (Lat.): refers to the species being found in subtropical area.

##### Description.

***Basidiomata*.** Annual, resupinate, inseparable from the substrate, without odor or taste and corky when fresh, rigid when dry, up to 15 cm long, 8 cm wide and 2.5 mm thick at center. Pore surface grayish brown to honey-yellow; sterile margin indistinct, curry-yellow, up to 1 mm wide; pores irregular to angular, sometimes sinuous, 3–5 per mm; dissepiments thin, entire, abundant setae seen in tube cavities (under lens). Subiculum clay-buff, corky, about 0.5 mm thick. Tubes olivaceous buff, up to 2 mm long.

**Figure 6.**
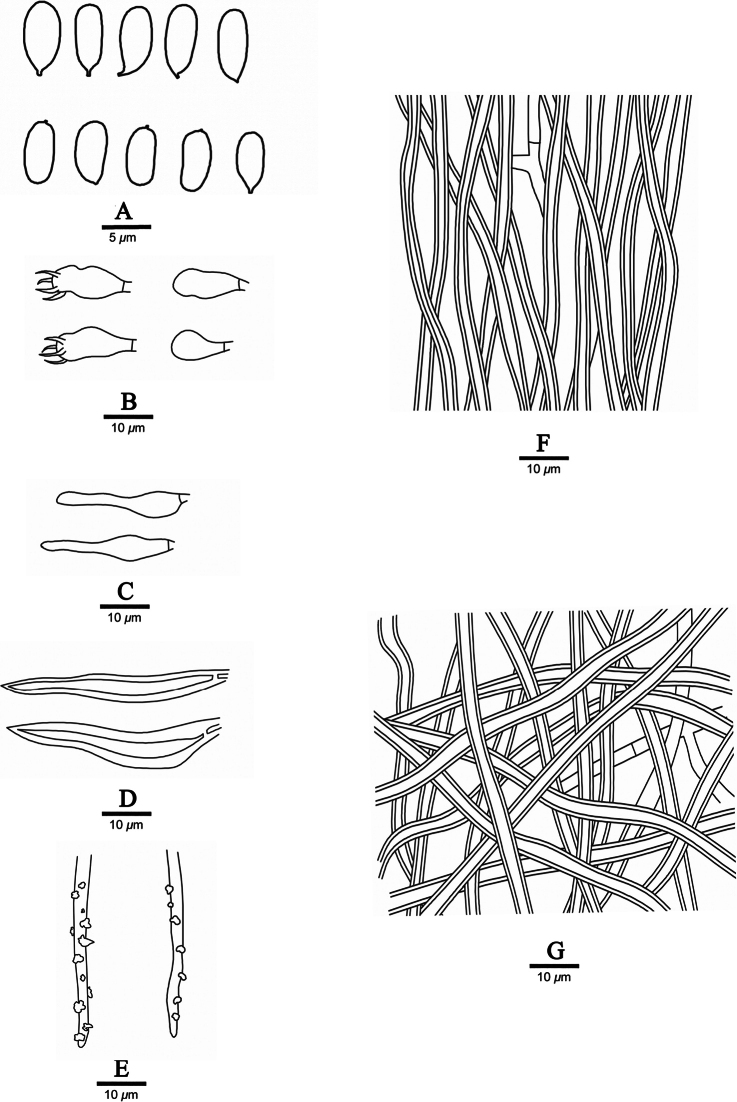
Microscopic structures of *Fuscoporiasubtropica* (holotype, Dai 19957) **A** basidiospores **B** basidia and basidioles **C** cystidioles **D** hymenial setae **E** generative hyphae at dissepiment edge **F** hyphae from tube trama **G** hyphae from subiculum.

***Hyphal structure*.** Hyphal system dimitic; generative hyphae simple septate; tissue becoming black in KOH.

***Subiculum*.** Generative hyphae infrequently, thin-walled, frequently branched, simple septate, 2–3 μm in diam; skeletal hyphae dominant, yellowish brown, thick-walled with a medium lumen, unbranched, aseptate, flexuous, strongly interwoven, 3–4 μm in diam.

**Figure 7.**
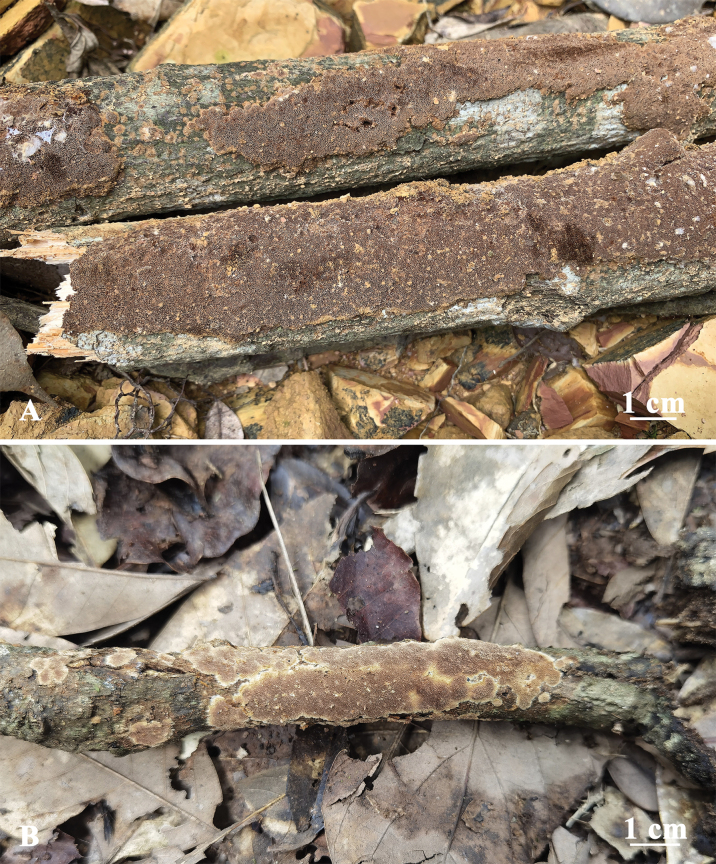
Basidiomata of *Fuscoporiasubtropica***A** Dai 19957 (holotype) **B** Dai 22604.

***Tubes*.** Generative hyphae infrequent, mostly present at subhymenium, hyaline, thin-walled, frequently branched, simple septate, 2–3 μm in diam; skeletal hyphae dominant, yellowish brown, thick-walled with a narrow to medium lumen, unbranched, aseptate, loosely interwoven, 2–4 μm in diam. Setae frequent, mostly originating from hymenium, subulate, dark brown, thick-walled, 35–55 × 4–7 μm; fusoid cystidioles frequent, hyaline and thin-walled, 18–26 × 4–6 μm; basidia barrel-shaped, with four sterigmata and a simple septum at the base, 14–18 × 4–6 μm; basidioles in shape similar to basidia, but slightly smaller.

***Basidiospores*.** Basidiospores cylindric, hyaline, thin-walled, smooth, IKI–, CB–, (5.5–)6–7.5(–8) × 2–3(–3.2) μm, L = 6.91 μm, W = 2.66 μm, Q = 2.32–2.71 (n = 50/2).

##### Additional specimens examined.

China • Yunnan Province, Pu’er, Pu’er Forest Park, Xiniuping Scenic Area, on fallen angiosperm branch, 17 August 2019, Dai 20476 (BJFC 032144); • Taiyanghe National Forest Park, on fallen angiosperm branch, 8 July 2021, Dai 22604 (BJFC 037178).

## ﻿Discussion

*Fuscoporia* is a polypore genus causing wood decay, associated with angiosperms and gymnosperms (Dai et al. 2007; Wu et al. 2022a, b; Yuan et al. 2023; Zhao et al. 2024). The medicinal potential of *Fuscoporia*, such as *F.gilva* and *F.torulosa*, was confirmed by modern studies (Deveci et al. 2019; Wu et al. 2019; Duong and Dang 2022). *Fuscoporia* is widely distributed in Asia (Bakshi et al. 1970; Dai 2010; Chen and Dai 2019; Chen et al. 2019, 2020; Du et al. 2020), Africa (Reid 1975; Ryvarden and Johansen 1980; Chen et al. 2023b), Australia (Chen et al. 2020), Europe (Donk 1960; Ryvarden and Gilbertson 1994, Ryvarden and Melo 2017), South America and North America (Larsen and Cobb-Poulle 1990; Chen et al. 2019; Wu et al. 2022a, b; Chen et al. 2023a). In this study, three new species of *Fuscoporia* are described based on molecular analyses and morphological features in Australia and southern Asia.

The recent studies (Chen and Dai 2019; Chen et al. 2020) demonstrated that the species of *Fuscoporiaferrea* was a complex species. We recognized eight species in the group: *Fuscoporiaferrea* sensu stricto (Ryvarden and Gilbertson 1994; Lowe 1966) in the Northern Hemisphere, such as Northern China, Europe and North America; three new species reported in this study, *F.resupinata* and *F.subtropica* from southern Asia, *F.eucalypticola* from Australia; *F.ramulicola* (Chen and Dai 2019), *F.subferrea* (Chen and Yuan 2017) and *F.yunnanensis* (Dai 2010) also distribution in south China; *F.punctatiformis* in Neotropics (Spirin et al. 2006), such as Brazil and USA. Eight species clustered into a clade with high statistical support (100/1.00/100) in phylogenetic analysis published in this study. The members of the *Fuscoporiaferrea* group differ from other species in the genus by its resupinate basidiomata, presence of hymenial setae and cystidioles, absence of mycelial setae, and cylindric basidiospores (Dai 2010; Chen and Yuan 2017; Chen and Dai 2019).

The species in the *Fuscoporiaferrea* group have similar morphological characteristics, which sometimes may be confused. However, *F.ferrea* and *F.punctatiformis* can be segregated from the three new species by their perennial basidiomata (Lowe 1966; Spirin et al. 2006). The remaining species of the *F.ferrea* group have annual basidiomata and are distributed in southern Asia, except for *F.eucalypticola*, which is from Australia and grows on *Eucalyptus.* Furthermore, two samples of *F.eucalypticola* formed a well-supported lineage (100/1.00/100), indicating that they are phylogenetically distinct from other species in Fig. 1. *Fuscoporiaeucalypticola* is closely related to *F.subtropica* in the phylogenetic tree and also has similar macromorphology in sharing annual basidiomata, irregular to angular, sometimes sinuous and bigger porse (3–5 per mm), but the latter differs in being without guttule in basidiospores and its distribution in Yunnan provinces, China.

Southern Asia is among the regions with the highest fungal biodiversity, especially in southern China (Dai et al. 2021; Zhou et al. 2023). *Fuscoporiasubtropica*, *F.yunnanensis*, *F.ramulicola* and *F.resupinata*are distributed in Yunnan provinces, China, *F.resupinata*are also distributed in Malaysia, *F.subferrea* is distributed in Hainan provinces, China, which is an island. Macromorphologically the two new species, *Fuscoporiaresupinata* and *F.subtropica*, are also similar to *F.subferrea*, *F.ramulicola* and *F.yunnanensis*, but *F.resupinata* differs from *F.yunnanensis* and *F.subferrea* by its medium-sized pores (5–7 per mm in *F.resupinata* vs. 3–4 per mm in *F.yunnanensis*, 7–10 per mm in *F.subferrea*; Chen and Yuan 2017); differs from *F.ramulicola* by its wider spores (2.5–3 μm, Q = 2.29–2.44 in *F.resupinata* vs. 2–2.5 μm, Q = 2.57–2.88 in *F.ramulicola*; Chen and Dai 2019). *Fuscoporiasubtropica* differs from *F.ramulicola* and *F.subferrea* by its larger pores (3–5 per mm in *F.subtropica* vs. 6–7 per mm in *F.ramulicola*, 7–10 per mm in *F.subferrea*), differs from *F.yunnanensis* by its irregular pores (Dai 2010). *Fuscoporiaresupinata* resembles *F.subtropica* by having annual and resupinate basidiomata, cylindric spores, but the former has smaller pores (5–7 per mm vs. 3–5 per mm), shorter fusoid cystidioles (8–12 μm vs. 18–26 μm), and its basidiospores sometimes with guttules.

### ﻿A key to resupinate and mycelial setaeless species of *Fuscoporia* in the world

**Table:** 

1	Basidiomata perennial	**2**
–	Basidiomata annual to biennial	**4**
2	Basidiospores narrowly ovoid to narrow ellipsoid	***F.montana* Y.C. Dai & Niemela**
–	Basidiospores cylindric to subcylindrical	**3**
3	Basidiospores 4–6 × 1.5–2 μm	***F.punctatiformis***
–	Basidiospores 6–7.8 × 2–2.5 μm	***F.ferrea***
4	Pores 3–5 per mm	**5**
–	Pores 5–10 per mm	**7**
5	Pores circular, dissepiments entire and matted	***F.yunnanensis***
–	Pores sinuous or irregular or daedaleoid, dissepiments entire and slightly lacerate with age	**6**
6	Basidiospores without guttule, Q = 2.32–2.71, distribution in China	***F.subtropica***
–	Basidiospores occasionally with a small guttule, Q = 2.86–3.05, distribution in Australia	***F.eucalypticola***
7	Pores 7–10 per mm; basidiospores 4.2–6.2 μm long, Q = 2.15–2.27	***F.subferrea***
–	Pores 5–7 per mm, basidiospores 5.5–7 μm long, Q > 2.27	**8**
8	Basidiospores 2.5–3 μm wide, Q = 2.29–2.44	***F.resupinata***
–	Basidiospores 2–2.5 μm wide, Q = 2.57–2.88	***F.ramulicola***

## Supplementary Material

XML Treatment for Fuscoporia eucalypticola

XML Treatment for Fuscoporia resupinata

XML Treatment for Fuscoporia subtropica

## ﻿Additional information

### Author ORCIDs

Qian Chen https://orcid.org/0000-0001-7355-714X

Han Chen https://orcid.org/0009-0001-8424-9916

Cheng-Hang Luo https://orcid.org/0009-0005-6059-6373

Xiao-Hong Lai https://orcid.org/0000-0001-5393-4307

### Data availability

All of the data that support the findings of this study are available in the main text.
